# Genetic architecture of type 1 diabetes with low genetic risk score informed by 41 unreported loci

**DOI:** 10.1038/s42003-021-02368-8

**Published:** 2021-07-23

**Authors:** Hui-Qi Qu, Jingchun Qu, Jonathan Bradfield, Luc Marchand, Joseph Glessner, Xiao Chang, Michael March, Jin Li, John J. Connolly, Jeffrey D. Roizen, Patrick Sleiman, Constantin Polychronakos, Hakon Hakonarson

**Affiliations:** 1 The Center for Applied Genomics, Children’s Hospital of Philadelphia, Philadelphia, PA USA; 2 Quantinuum Research LLC, San Diego, CA USA; 3 Department of Pediatrics, McGill University, Montreal, QC Canada; 4 Department of Cell Biology, Tianjin Medical University, Tianjin, China; 5 Department of Pediatrics, The Perelman School of Medicine, University of Pennsylvania, Philadelphia, PA USA; 6 Division of Human Genetics, Children’s Hospital of Philadelphia, Philadelphia, PA USA; 7 Centre of Excellence in Translational Immunology, Research Institute of McGill University Health Centre, Montreal, QC Canada; 8 Division of Pulmonary Medicine, Children’s Hospital of Philadelphia, Philadelphia, PA USA

**Keywords:** Molecular medicine, Diabetes

## Abstract

Type 1 diabetes (T1D) patients with low genetic risk scores (GRS) may be non-autoimmune or autoimmune mediated by other genetic loci. The T1D-GRS2 provides us an opportunity to look into the genetic architecture of these patients. A total of 18,949 European individuals were included in this study, including 6599 T1D cases and 12,323 controls. 957 (14.5%) T1D patients were identified with low GRS (GRS < 8.43). The genome-wide association study on these patients identified 41 unreported loci. Two loci with common variants and 39 loci with rare variants were identified in this study. This study identified common SNPs associated with both low GRS T1D and expression levels of the interferon-α-induced *MNDA* gene, indicating the role of viral infection in T1D. Interestingly, 16 of the 41 unreported loci have been linked to autism spectrum disorder (ASD) by previous studies, suggesting that genes residing at these loci may underlie both T1D and autism.

## Abstract (web summary)

Hui-Qi Qu et al. present a case-control association study to examine the genetic architecture of European patients with type 1 diabetes (T1D) of low genetic risk score. They identify 41 loci associated with T1D of low genetic risk score, including regions previously linked to viral infection and autism spectrum disorder, altogether providing further insight into the pathogenesis of T1D.

## Introduction

Type 1 diabetes (T1D) has been traditionally recognized as an autoimmune disease, and its molecular immunological mechanisms have been corroborated by the discovery of numerous autoimmune disease genes underlying the genetic susceptibility of T1D^1–3^. As we showed in a recent study^4^, the genetic risk of T1D can be predicted by genome-wide DNA variants generated using a global polygenic risk scoring (PRS) approach^5^. In contrast, Sharp et al. developed a specific genetic risk scoring (GRS) system for T1D, (T1D-GRS2), using 67 single nucleotide polymorphisms (SNPs) from known autoimmune loci associated with T1D, while haplotypic effects and interactions of common human leukocyte antigen (*HLA*) *DR-DQ* haplotypes, conferring the primary effects to T1D susceptibility^1,6–8^, were also taken into account^9^. In contrast to global PRS scoring using genome-wide DNA markers, T1D-GRS2 uses a small set of T1D genetic markers. Among the 67 SNPs, 35 are from the major T1D susceptibility loci, i.e., the *HLA* region accounting for about 50% of the genetic susceptibility in the European population^1^. The other 32 SNPs are from 31 non-HLA T1D susceptibility loci.

T1D is a complex and heterogeneous phenotype. A minor proportion (~5–10%) of Caucasian patients diagnosed with T1D have non-autoimmune pathogenesis, i.e., T1bD^10^. Moreover, there are also autoimmune patients (e.g., with islet cell auto-antibodies), but with low-risk genotypes of known T1D genes, e.g., protective *HLA* haplotypes^11^. The PRS approach using genome-wide DNA markers represents primarily autoimmune T1D^4^, whereas low PRS suggests non-autoimmune mechanisms may be involved. As expected, a number of loci identified in our analysis have been reported of association with obesity-related traits by previous GWA studies^4^. Our gene-based association study in patients with low PRS identified the Notch ligand Delta-like 1 gene (*DLL1*)^12^, involved in impaired glucose tolerance and reduced insulin secretion^13^. In contrast, low GRS may also include autoimmune patients with undetermined genetic mechanisms. Thus, patients with T1D and low GRS may have their disease susceptibility conferred by other unknown genetic loci. Due to these potentially distinct biological mechanisms underlying T1D captured by PRS vs. GRS, respectively, the T1D-GRS2 scoring system provides us a unique opportunity to identify patients with low GRS and consequently enables us to look into the genetic architecture of this group of patients.

## Results

### GRS scores

As shown by our ROC analysis, the GRS scores for T1D prediction have the performance of the Area Under the ROC Curve (AUC) = 0.866. The cutoff of GRS = 8.43 has the maximum Matthews correlation coefficient (MCC) of 0.580, with the sensitivity to identify T1D patients (true positive rate, TPR) of 0.855 and the specificity to identify individuals without T1D (true negative rate, TNR) of 0.719 (Supplementary Data 2). We identified 957 (14.5%) T1D patients with GRS < 8.43.

In this study, the GRS scores are significantly correlated with the PRS scores (*P* < 1E − 200), with Pearson’s *r* = 0.305 and 0.331, respectively in the two PRS cohorts, as we reported^4^. Among the 6599 T1D cases, 4314 (65.4%) cases have both high GRS and high PRS scores; 381 (5.8%) cases have both low GRS and low PRS scores; 1328 (20.1%) cases have high GRS and low PRS; and 576 (8.7%) cases have low GRS and high PRS.

### GWAS of T1D patients with low GRS

**Fig. 1:**
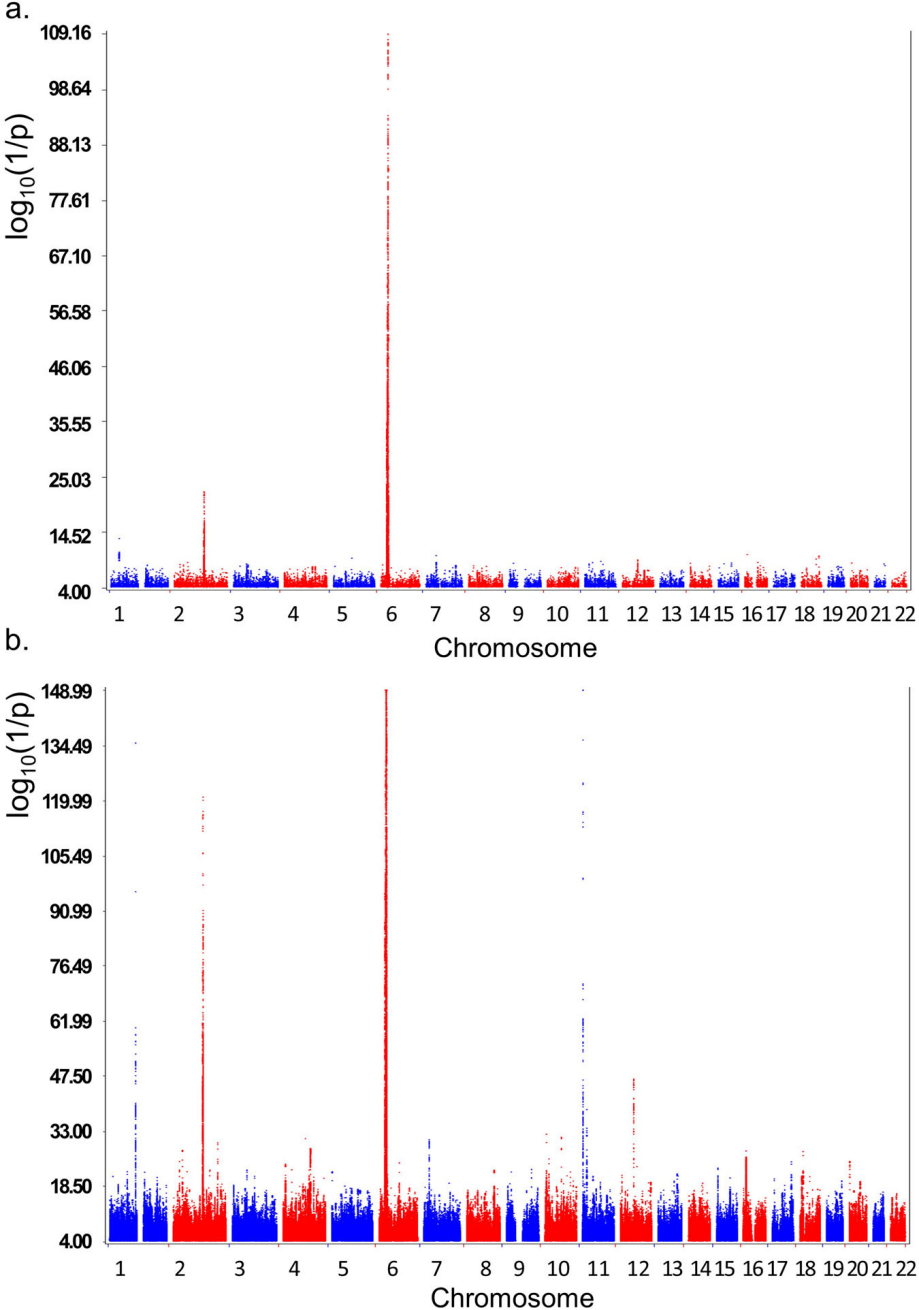
The Manhattan plots of this study. **a** the GWAS of T1D patients with low T1D GRS (957 cases vs. 12,350 controls); (**b**) The plot of the GWAS of all T1D patients (6599 cases vs. 12,350 controls).

Nine hundred and fifty-seven T1D patients (474 males and 483 females) were identified with low GRS. The *HLA* loci contributed significantly to this GRS classification (Table 1), while the *HLA-DQ* locus contributes more to the classification (beta = 0.489) than the combined effects of other *HLA* loci (beta = 0.308). The GWAS results of patients with low T1D GRS *vs*. all non-diabetes controls (Fig. 1a) were compared with that of all T1D patients vs. all non-diabetes controls (Fig. 1b). As expected, the majority of SNPs showing genome-wide significance in low T1D GRS were also significant in the overall T1D cohort (Supplementary Data 3), which is in keeping with our original hypothesis that low GRS cases may also be driven by autoimmune mechanisms. However, the potential undetermined genetic mechanisms of the low T1D GRS patients are highlighted by 82 single nucleotide variants (SNV) from 47 non-*HLA* genetic loci with genome-wide significance in the low GRS T1D cases only, but not significant in the overall T1D cohort (Supplementary Data 4). The minor alleles of 81 out of the 82 SNVs are predisposing, except the SNP rs62425513 at the *THEMIS* locus. Without exception, however, the genetic effects represented by these SNPs in the low GRS T1D cases are all greater than the effects observed in the overall T1D cohort, where each SNP’s OR in the overall T1D cohort fell outside the 95% confidence interval (95% CI) in the low GRS T1D cohort, although 11 SNVs from 8 loci have the two-tailed P values of heterogeneity test >0.05.

**Table 1:** Contribution of the *HLA* loci to the GRS classification.

Locus	T1D	*N*	Mean^a^	Std. deviation	*P*
*HLA-DQ*	Low GRS	951	−0.348	0.977	<1E − 200
	High GRS	5625	2.052	1.462	
Other *HLA*	Low GRS	957	3.984	0.991	8.85E − 191
	High GRS	5642	4.999	0.946	
All *HLA*	Low GRS	957	3.638	1.209	<1E − 200
	High GRS	5642	7.044	1.657	

^a^Aggregate effects. The gene name *HLA* is in italics.

### Unreported loci with common variants associated with low GRS T1D

**Fig. 2:**
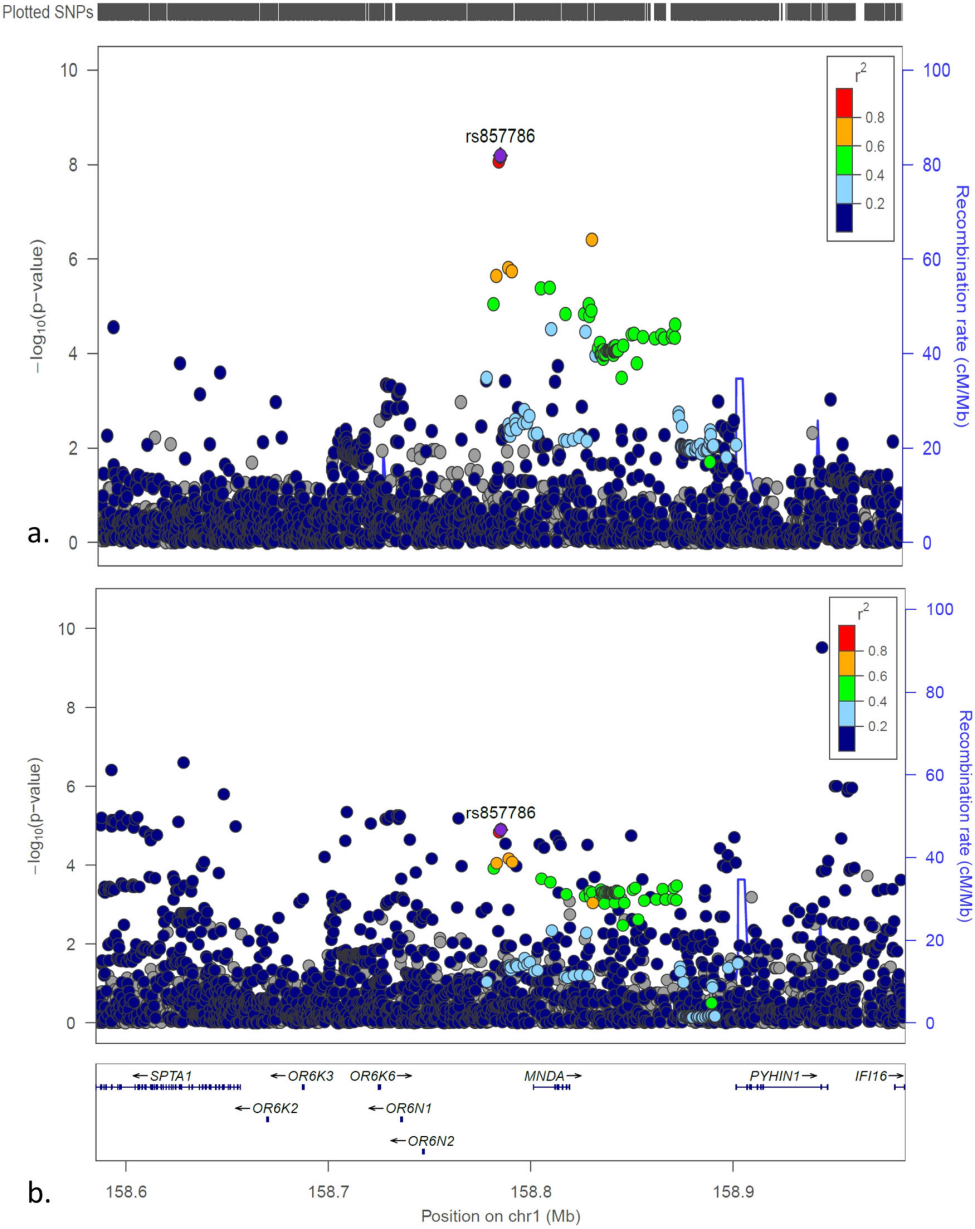
The LocusZoom plots for the *OR6N2/MNDA/PYHIN1* locus. **a** The plot of the association tests of T1D patients with low T1D GRS compared to controls; (**b**) The plot of the association tests of all T1D patients compared to controls.

**Fig. 3:**
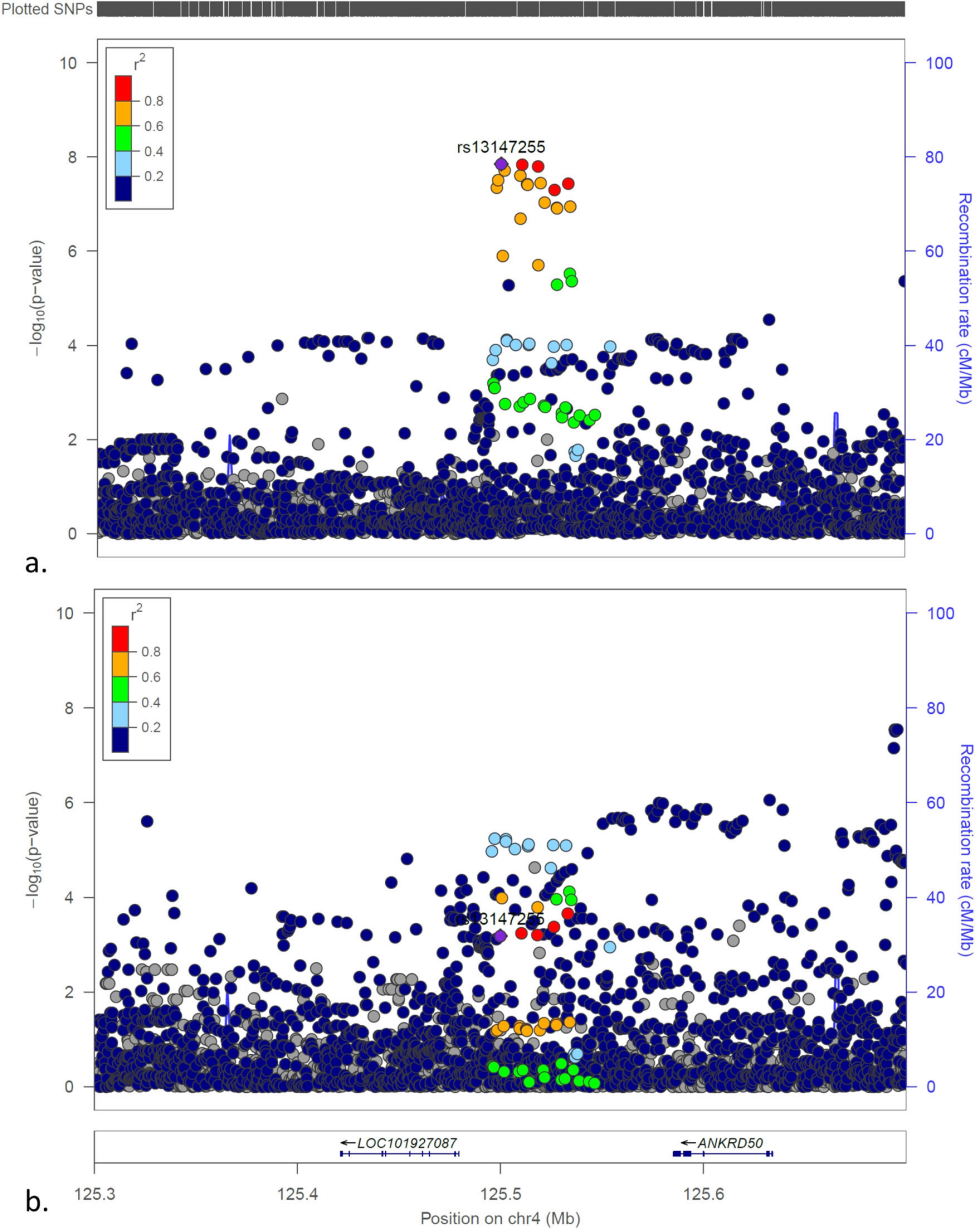
The LocusZoom plots for the *LINC02516/ANKRD50* locus. **a** The plot of the association tests of T1D patients with low T1D GRS compared to controls; (**b**) The plot of the association tests of all T1D patients compared to controls.

Among the 82 genome-wide significant SNPs, 19 common SNPs from 5 independent genetic loci have minor allele frequencies (MAF) > 0.050. 16 common SNPs from 2 genetic loci have MAF in the range of 0.304 to 0.462. (1) The locus at chr1q23.1 (Fig. 2) harbors several coding genes, including the myeloid cell nuclear differentiation antigen gene (*MNDA*), encoding an interferon-inducible gene, and the pyrin and HIN domain family member 1 gene (*PYHIN1*), encoding an interferon-inducible gene. The strongest association signal in low T1D GRS (Fig. 2a), which is lack significance in all T1D patients (Fig. 2b), is tagged by the SNP rs857786 upstream of *MNDA*, with OR (95% CI) = 1.322 (1.203, 1.452), *P* = 6.44E − 09, representing a strong effect size for a common SNP. (2) The association signal in low T1D GRS [Fig. 3a, lack of significance in all T1D patients (Fig. 3b)], tagged by the SNP rs13147255 with OR(95% CI) = 1.318(1.198,1.449), *P* = 1.42E − 08, at the chr4q28.1 locus, resides between the long intergenic non-protein coding RNA 2516 gene (*LINC02516*) and the ankyrin repeat domain 50 genes (*ANKRD50*).

### Unreported loci with rare variants associated with low GRS T1D

Among the 42 independent loci with rare variants (MAF < 0.050), one locus (i.e., the *PGM1* locus) has been reported of association with T1D by previous GWAS studies (Supplementary Data 5, GWAS Catalog, https://www.ebi.ac.uk/gwas/) and two recent large-scale T1D GWAS^14,15^, thus was not taken as an unreported locus. In addition, the *DOK6* locus identified in this study, led by the SNV rs146427450 with OR(95% CI) = 2.858(2.076, 3.935), *P* = 1.22E − 10, is close to (~227 kb) the *CD226* locus identified by the studies by Robertson et al.^15^ and Crouch et al.^14^ Another locus tagged by the SNV rs148505224 at *CFTR* with OR(95% CI) = 3.107(2.090, 4.619), *P* = 2.09E − 08, is close to the *ASZ1* locus recently identified by Crouch et al.^14^ Besides these 3 loci and the above loci with common genetic variants associated with low GRS T1D, we uncovered 39 unreported loci with variants in the low to rare frequency range (MAF ≤ 3.92% in this study) associated with low GRS T1D (Supplementary Data 4).

## Discussion

**Fig. 4:**
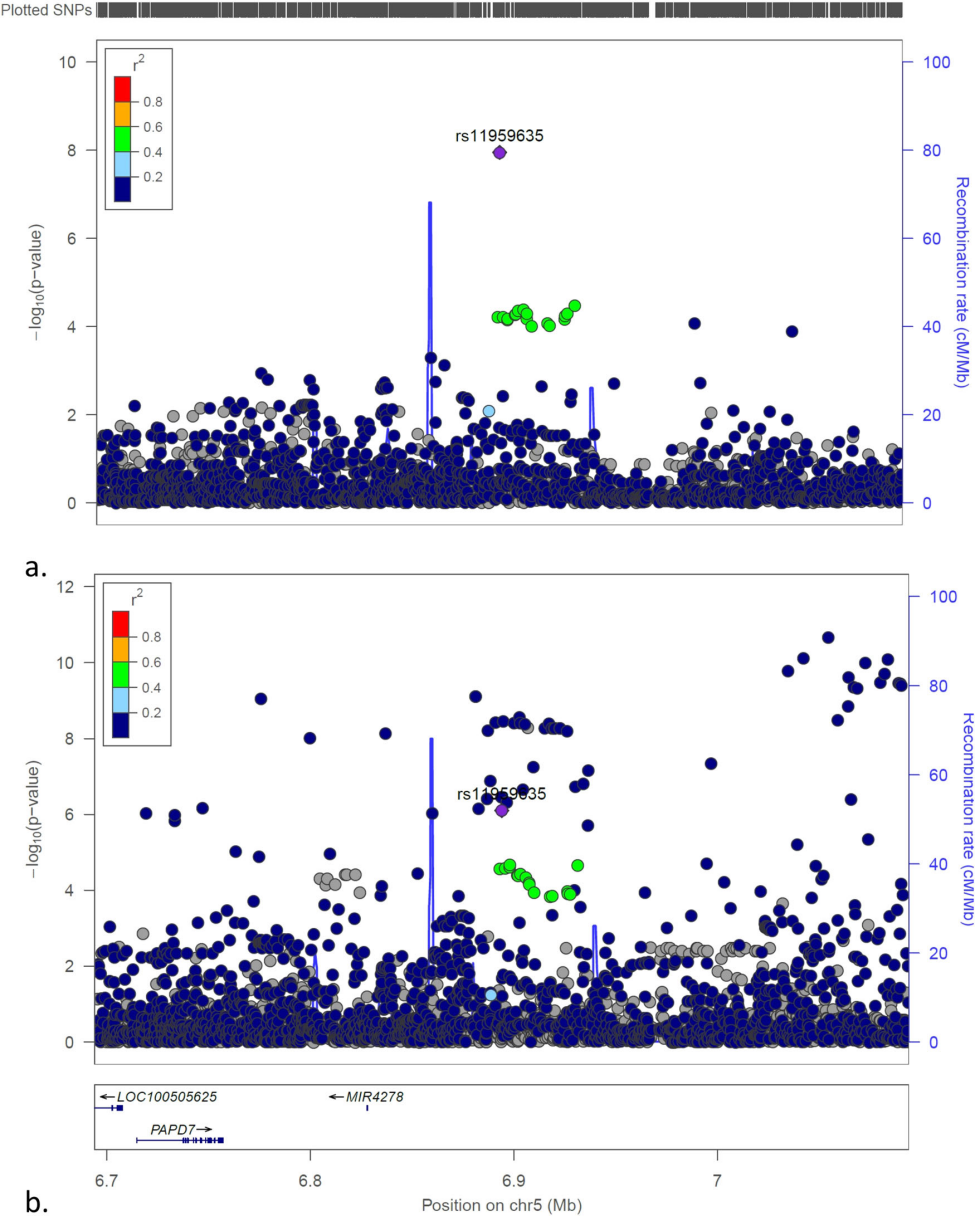
The LocusZoom plots for the *MIR4278;MIR4454* locus. **a** The plot of the association tests of T1D patients with low T1D GRS compared to controls; (**b**) The plot of the association tests of all T1D patients compared to controls.

**Fig. 5:**
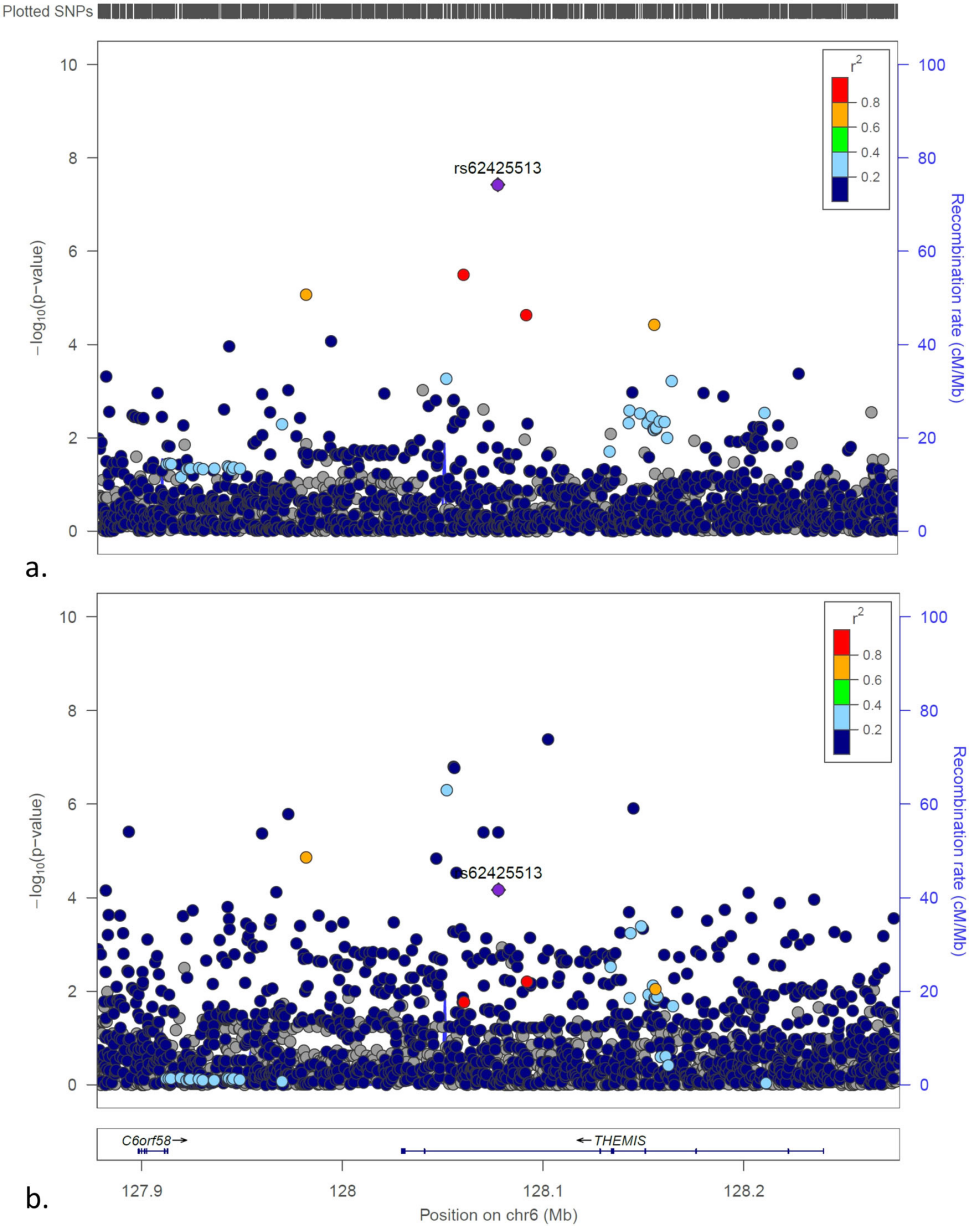
The LocusZoom plots for the *THEMIS* locus. **a** The plot of the association tests of T1D patients with low T1D GRS compared to controls; (**b**) The plot of the association tests of all T1D patients compared to controls.

**Fig. 6:**
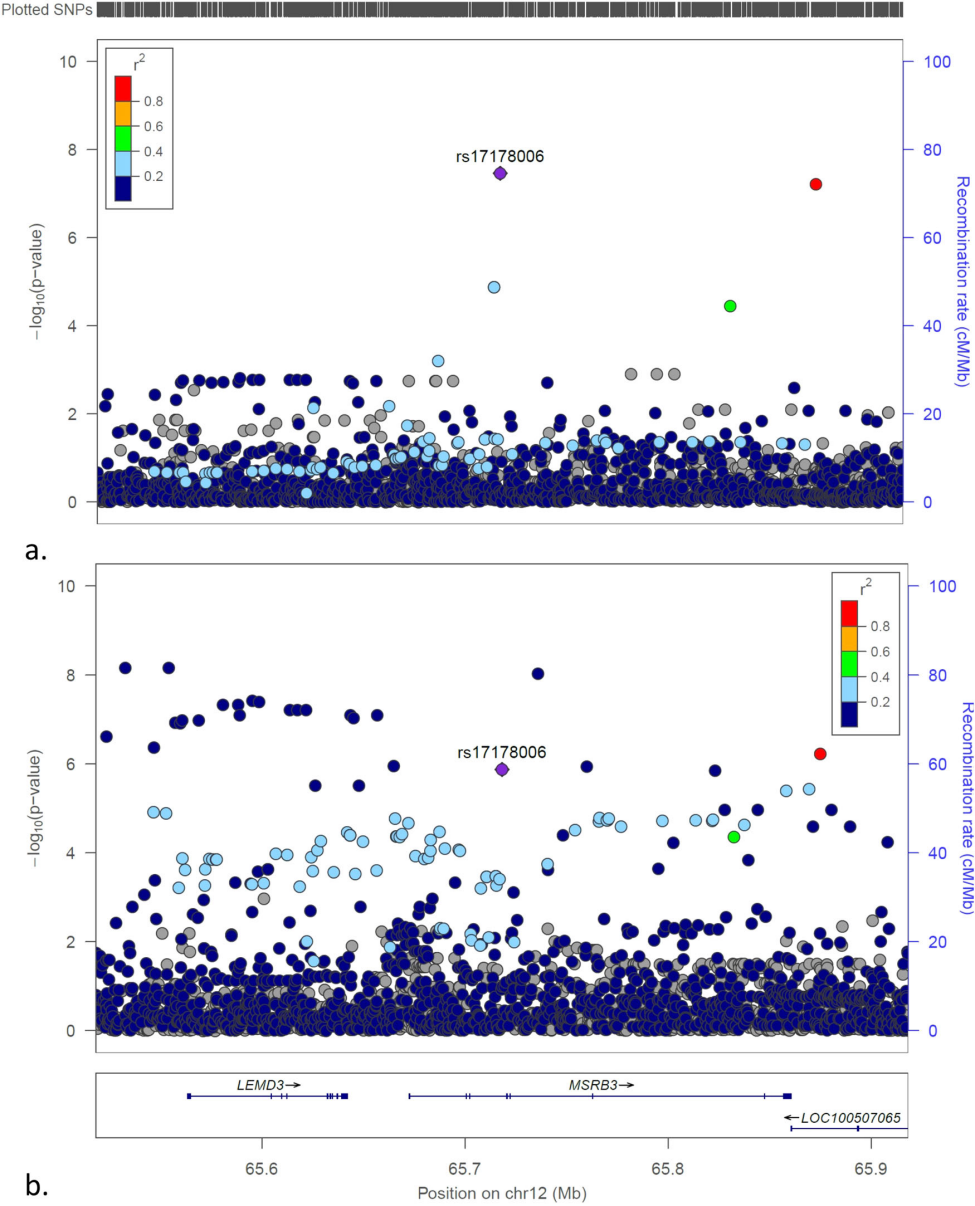
The LocusZoom plots for the *MSRB3* locus. **a** The plot of the association tests of T1D patients with low T1D GRS compared to controls; (**b**) The plot of the association tests of all T1D patients compared to controls.

Five loci with common variants were identified of association with low GRS T1D in this study with genome-wide significance. Besides the above 2 loci with common variants, 3 SNPs from 3 different loci (*MIR4278/MIR4454, THEMIS, MSRB3*) have also common SNPs with minor allele frequencies (MAF) > 0.050 associated with low GRS T1D (Figs. 4a, 5a, 6a), but not the general T1D cases (Figs. 4b, 5b, 6b). However, as shown in Figs. 4–6, different SNPs at each loci are associated with the general T1D cases, therefore these 3 loci were not taken as unreported loci specifically associated with low GRS T1D in this study. In addition, the *THEMIS* locus tagged by the SNP rs62425513 with OR (95% CI) = 0.673(0.584,0.775), *P* = 3.76E − 08, was identified of T1D association with FDR < 0.01 by Robertson, et al.^15^. Interestingly, each of these 3 loci has been identified of association with obesity-related traits or waist-hip ratio by the previous GWASs (Supplementary Data 5). The *THEMIS* locus was also reported of association with celiac disease by the previous GWAS^16^.

The unreported T1D genetic locus *MNDA* is involved in interferon signaling. At the *OR6N2/MNDA/PYHIN1* locus, the strongest association signal rs857786 with OR(95% CI) = 1.322(1.203,1.452), *P* = 6.44E − 09, at the 5′-upstream of *MNDA*, is also associated with the gene expression of *MNDA* in whole blood (*P* = 9.9e − 14), according to the expression quantitative trait loci (eQTLs) data of the GTEx Project (https://www.gtexportal.org/)^17^. The protein encoded by *MNDA* is expressed specifically in hematopoietic cells, and upregulated by interferon-α^18^. Viral infections have been suggested as a possible trigger of T1D, although the evidence remains controversial^19,20^. Interferon-α is a potential link of viral infection and autoimmunity in T1D^21^. In our study, significant association from the interferon-α-induced *MNDA* locus is only seen in low GRS cases, but not in overall T1D cases (Fig. 2). This may imply a plausible explanation about the pathogenesis of low GRS T1D patients, i.e., that viral infection contributes to the T1D pathogenesis in these patients despite the low overall genetic risk. In addition to our study, a previous study has shown an association of this locus with monocyte chemoattractant protein-1 levels^22^.

The unreported T1D genetic locus *LINC02516/ANKRD50* is involved in retromer function. *ANKRD50* has been demonstrated of essential role in the function of retromer and the endocytic recycling^23^. The protein encoded by *ANKRD50* is an essential component for the retromer function^23^. The retromer mediates the retrograde transport from the endosome to the Golgi^24^. The retromer protein VPS35 which mediates the retromer cargo selection has been shown to be associated with T2D in a previous GWAS study^25^. The gene encoding a receptor of VPS35, the sortilin related VPS10 domain-containing receptor 1 gene (*SorCS1*), has also been reported in association with glycemic control in T1D^26^ and insulin secretion in T2D^27^.

Association between autism and T1D has been reported previously^28^. Interestingly, 15 of the 39 unreported loci identified of genome-wide significance in this study have been reported to harbor variants predisposing to autism or autism spectrum disorder (ASD) according to the HGMD database (http://www.hgmd.cf.ac.uk) (Supplementary Data 4), and 2 of these loci have been previously reported of association with ASD by the previous GWASs^29,30^. Six genes at these loci are expressed at the cell synapse (cellular_component GO:0045202), including ankyrin 3 (*ANK3*), cell cycle associated protein 1 (*CAPRIN1*), cadherin 8 (*CDH8*), fibroblast growth factor receptor 2 (*FGFR2*), olfactomedin 3 (*OLFM3*), and prion protein (*PRNP*). Mechanisms of neural control of the endocrine pancreas^31^ mediated by these genes are therefore highlighted. Besides the 15 loci linked to autism or ASD, two other loci with rare variants associated with low GRS T1D encode synapse-expressed genes, abhydrolase domain containing 17B (*ABHD17B*) and leucine-rich repeat and fibronectin type III domain containing 3 (*LRFN3*). It is also worth mentioning that, at the *OR6N2/MNDA/PYHIN1* locus with common SNPs associated with low GRS T1D, the pyrin and HIN domain family member 1 gene (*PYHIN1*) whose expression is also interferon-induced has also been linked to autism and ASD by the previous study^32^.

The SNV rs148505224 associated with low GRS T1D maps to the CF transmembrane conductance regulator (*CFTR*) gene, i.e., the gene mutated in cystic fibrosis(CF) and a cause of pancreatitis. As this *CFTR* locus is close to the *ASZ1* locus recently identified by Crouch et al.^14^, it is not counted as an unreported locus identified in this study. The chr4p15.2 locus encoding the peroxisome proliferator-activated receptor gamma (PPAR-γ) coactivator 1 alpha gene (*PPARGC1A*), which has been shown to important roles in energy homeostasis^33^. Besides the 3 loci with common SNPs associated with waist-to-hip ratio or obesity-related traits, 8 genetic regions with rare variants demonstrating an association with low GRS T1D have been previously reported in association with body mass index (BMI), weight, waist-hip ratio, or obesity-related traits (Supplementary Data 5).

In addition to the *MNDA* locus, two loci have been reported of association with severe influenza A (H1N1) infection, including the locus at chr2q14.1 encoding the dipeptidyl peptidase like 10 genes (*DPP10*) and the locus at chr19q13.12 containing the nuclear factor kappa B (NF-κB) inhibitor delta gene (*NFKBID*)^34^. *NFKBID* is critical for B cell development as shown in mouse model^35^. In addition to the association of *THEMIS* with celiac disease, two loci with rare variants have been reported of association with autoimmune diseases, i.e., the long intergenic non-protein coding RNA 1967 (*LINC01967*) and C-X9-C motif containing 1 (*CMC1*) locus at chr3p24.1 has been reported to be associated with multiple sclerosis^36^, and the small nucleolar RNA, C/D box 3F (*SNORD3F*)/leucine zipper tumor suppressor 1 (LZTS1) antisense RNA 1 (*LZTS1-AS1*) locus at chr8p21.3 which has been reported to be associated with rheumatoid arthritis^37^.

In conclusion, this study identified 41 unreported loci associated with low GRS T1D. In addition to our previous study on low PRS T1D which identified new non-autoimmune T1D loci^4^, this study identified common genetic variants at two loci related to interferon signaling involved in viral infection and retromer function, respectively. The role of viral infection in low GRS T1D is supported by the common SNPs associated with low GRS T1D inferring considerable effect size (OR~1.32). Likewise, unreported loci related to pancreatitis, BMI, and obesity were also uncovered. 16 of 41 loci have been previously linked to autism or ASD^38,39^ (http://www.hgmd.cf.ac.uk). Accordingly, this study highlights a number of genes that may mediate shared molecular mechanisms of ASD and T1D. From the molecular genetics aspect, these rare variants may suggest a possibility of rare syndromic types of disease with clinical characterizations of both ASD and T1D not previously identified. Additional studies are needed to confirm our hypothesis and preliminary results, that patients with both ASD and T1D diagnosis may share common genetic factors. Patients with low T1D GRS may also be autoimmune, i.e., T1aD with undetermined genetic mechanisms, or non-autoimmune, e.g., T1bD. Among the genetic loci identified in this study, 3 loci with common SNPs and 8 genetic regions with rare variants have been previously reported in association with BMI, waist-to-hip ratio, or obesity-related traits, suggesting T1bD. It is also worth pointing out that these reported variants may still be autoimmune T1D loci, suggested by the two loci reported of association with autoimmune diseases, and the two loci associated with severe influenza A infection. Patients with low T1D GRS, whether they are T1aD or not, may have their disease susceptibility conferred by these loci with inflated effect sizes in this subgroup of patients.

## Methods

### Subjects

A total of 18,949 European individuals were included in this study, including 6599 T1D cases and 12,323 controls. The T1D cases were from Montreal Children’s Hospital and the Children’s Hospital of Philadelphia (CHOP)^3^, The Diabetes Control and Complications Trial – Epidemiology of Diabetes Interventions and Complications (DCCT-EDIC) cohort (http://www.ncbi.nlm.nih.gov/projects/gap/cgi-bin/study.cgi?study_id=phs000086.v2.p1), and the Type 1 Diabetes Genetics Consortium (T1DGC, http://www.ncbi.nlm.nih.gov/projects/gap/cgi-bin/study.cgi?study_id=phs000180.v1.p1), respectively. Informed consent was obtained from each of the relevant cohorts/studies. The genotyping was done by the Illumina Genotyping BeadChips with at least 550,000 SNPs genotyped. More details of these research subjects are previously described^3,40^. All the research subjects have been confirmed of European ancestry by principal component analysis (PCA) with genomic DNA markers. Genome-wide imputation was done by the TOPMed Imputation Server (https://imputation.biodatacatalyst.nhlbi.nih.gov) with the TOPMed (Version R2 on GRC38) Reference Panel.

### GRS scoring

The GRS scoring was based on the method developed by Sharp et al.^9^. To acquire the genotype information of all the T1D-GRS2 SNPs (67 SNP markers, Supplementary Data 1), the *HLA* region was additionally imputed by the SNP2HLA software^41^. The overlapped SNPs covered across the imputation methods were highly consistent. Consequently, the GRS scores were assessed for their predictive performance by AUC. The GRS cutoff for low GRS vs. high GRS was determined by the maximum MCC, which represents a balanced measure of sensitivity and specificity.

### Statistics and reproducibility

We tested 104,689,647 autosomal SNV with quality filters of *R*^2^ ≥ 0.3 for the genetic association, using 12,323 controls (6665 males and 5658 females) and 6599 T1D cases, including 957 T1D patients (474 males and 483 females) with low GRS. Genetic association tests were performed using PLINK1.9 software^42^, conditioned on sex, and corrected by the first 10 principal components (PC) of population structure analysis. Genome-wide significance was defined as *P* < 5 × 10^−8^. The Manhattan plots were done using the SNPEVG software^43^. Genetic association signals within each locus were plotted by LocusZoom^44^. The genetic association of T1D patients with low GRS was compared with that of the general T1D patients by heterogeneity *Z* test^45^. We defined each locus by *r*^2^ > 0.5 from lead SNPs^46^.

### Reporting summary

Further information on research design is available in the Nature Research Reporting Summary linked to this article.

## Supplementary information

Description of Supplementary Files

Supplementary Data 1

Supplementary Data 2

Supplementary Data 3

Supplementary Data 4

Supplementary Data 5

Reporting Summary

## Data Availability

Summary statistics are presented in Supplementary Data 4 and are available at the NHGRI-EBI GWAS catalog (GCP ID: GCP000182, https://www.ebi.ac.uk/gwas). The original genotyping data are available from dbGAP. All data generated or analyzed during this study are included in this published article (and its supplementary information files).

## References

[1] Todd JA, Bell JI, McDevitt HO (1987). HLA-DQ[beta] gene contributes to susceptibility and resistance to insulin-dependent diabetes mellitus. Nature.

[2] Wellcome Trust Case Control Consortium. (2007). Genome-wide association study of 14,000 cases of seven common diseases and 3,000 shared controls. Nature.

[3] Hakonarson H (2007). A genome-wide association study identifies KIAA0350 as a type 1 diabetes gene. Nature.

[4] Qu, J. et al. Genetics of low polygenic risk score type 1 diabetes patients: rare variants in 22 novel loci. *medRxiv* (2020).

[5] Choi SW, O’Reilly PF (2019). PRSice-2: polygenic risk score software for biobank-scale data. GigaScience.

[6] Todd JA (1995). Genetic analysis of type 1 diabetes using whole genome approaches. PNAS.

[7] Noble JA (1996). The role of HLA class II genes in insulin-dependent diabetes mellitus: molecular analysis of 180 Caucasian, multiplex families. Am. J. Hum. Genet..

[8] she J-X (1996). Susceptibility to type I diabetes: HLA-DQ and DR revisited. Immunol. Today.

[9] Sharp SA (2019). Development and standardization of an improved type 1 diabetes genetic risk score for use in newborn screening and incident diagnosis. Diabetes Care.

[10] Leslie RD, Atkinson MA, Notkins AL (1999). Autoantigens IA-2 and GAD in Type I (insulin-dependent) diabetes. Diabetologia.

[11] Noble JA, Valdes AM (2011). Genetics of the HLA region in the prediction of type 1 diabetes. Curr. Diabetes Rep..

[12] Qu J (2020). Association of DLL1 with type 1 diabetes in patients characterized by low polygenic risk score. Metabolism.

[13] Rubey M (2020). DLL1- and DLL4-mediated notch signaling is essential for adult pancreatic islet homeostasis. Diabetes.

[14] Crouch D. J. et al. Enhanced genetic analysis of type 1 diabetes by selecting variants on both effect size and significance, and by integration with autoimmune thyroid disease. *bioRxiv* (2021).

[15] Robertson C. C. et al. Fine-mapping, trans-ancestral and genomic analyses identify causal variants, cells, genes and drug targets for type 1 diabetes. *bioRxiv* (2020). 10.1038/s41588-021-00880-5 PMC8273124 34127860

[16] Coleman C (2016). Common polygenic variation in coeliac disease and confirmation of ZNF335 and NIFA as disease susceptibility loci. Eur. J. Hum. Genet..

[17] Lonsdale J (2013). The genotype-tissue expression (GTEx) project. Nat. Genet..

[18] Briggs RC (1994). The human myeloid cell nuclear differentiation antigen gene is one of at least two related interferon-inducible genes located on chromosome 1q that are expressed specifically in hematopoietic cells. Blood.

[19] Van der Werf N, Kroese FG, Rozing J, Hillebrands JL (2007). Viral infections as potential triggers of type 1 diabetes. Diabetes/Metab. Res. Rev..

[20] Filippi CM, von Herrath MG (2008). Viral trigger for type 1 diabetes: pros and cons. Diabetes.

[21] Devendra D, Eisenbarth G (2004). Interferon alpha—a potential link in the pathogenesis of viral-induced type 1 diabetes and autoimmunity. Clin. Immunol..

[22] Ahola-Olli AV (2017). Genome-wide association study identifies 27 loci influencing concentrations of circulating cytokines and growth factors. Am. J. Hum. Genet..

[23] Kvainickas A (2017). Retromer- and WASH-dependent sorting of nutrient transporters requires a multivalent interaction network with ANKRD50. J. Cell Sci..

[24] Trousdale C, Kim K (2015). Retromer: structure, function, and roles in mammalian disease. Eur. J. Cell Biol..

[25] Kooner JS (2011). Genome-wide association study in individuals of South Asian ancestry identifies six new type 2 diabetes susceptibility loci. Nat. Genet..

[26] Paterson AD (2010). A genome-wide association study identifies a novel major locus for glycemic control in type 1 diabetes, as measured by both A1C and glucose. Diabetes.

[27] Goodarzi MO (2007). SORCS1: a novel human type 2 diabetes susceptibility gene suggested by the mouse. Diabetes.

[28] Freeman SJ, Roberts W, Daneman D (2005). Type 1 diabetes and autism: is there a link?. Diabetes Care.

[29] Identification of risk loci with shared effects on five major psychiatric disorders: a genome-wide analysis. *Lancet* **381**, 1371–1379 (2013). 10.1016/S0140-6736(12)62129-1 PMC3714010 23453885

[30] Grove J (2019). Identification of common genetic risk variants for autism spectrum disorder. Nat. Genet..

[31] Rodriguez-Diaz R, Caicedo A (2014). Neural control of the endocrine pancreas. Best. Pract. Res. Clin. Endocrinol. Metab..

[32] Krumm N (2015). Excess of rare, inherited truncating mutations in autism. Nat. Genet..

[33] Esterbauer H, Oberkofler H, Krempler F, Patsch W (1999). Human peroxisome proliferator activated receptor gamma coactivator 1 (PPARGC1) gene: cDNA sequence, genomic organization, chromosomal localization, and tissue expression. Genomics.

[34] Garcia-Etxebarria K (2015). No major host genetic risk factor contributed to A(H1N1)2009 Influenza severity. PLoS ONE.

[35] Touma M (2011). Impaired B cell development and function in the absence of IkappaBNS. J. Immunol..

[36] International Multiple Sclerosis Genetics Consortium. Multiple sclerosis genomic map implicates peripheral immune cells and microglia in susceptibility. *Science* **365**, eaav7188 (2019). 10.1126/science.aav7188 PMC7241648 31604244

[37] Saad MN, Mabrouk MS, Eldeib AM, Shaker OG (2019). Studying the effects of haplotype partitioning methods on the RA-associated genomic results from the North American Rheumatoid Arthritis Consortium (NARAC) dataset. J. Adv. Res..

[38] Cross-Disorder Group of the Psychiatric Genomics Consortium. Genomic Relationships, Novel Loci, and Pleiotropic Mechanisms across Eight Psychiatric Disorders. *Cell* **179**, 1469–1482.e1411 (2019). 10.1016/j.cell.2019.11.020 PMC7077032 31835028

[39] The Autism Spectrum Disorders Working Group of The Psychiatric Genomics Consortium. Meta-analysis of GWAS of over 16,000 individuals with autism spectrum disorder highlights a novel locus at 10q24.32 and a significant overlap with schizophrenia. *Mol. Autism* **8**, 21 (2017). 10.1186/s13229-017-0137-9 PMC5441062 28540026

[40] Bradfield JP (2011). A genome-wide meta-analysis of six type 1 diabetes cohorts identifies multiple associated loci. PLoS Genet..

[41] Jia X (2013). Imputing amino acid polymorphisms in human leukocyte antigens. PloS ONE.

[42] Chang CC (2015). Second-generation PLINK: rising to the challenge of larger and richer datasets. Gigascience.

[43] Wang S, Dvorkin D, Da Y (2012). SNPEVG: a graphical tool for GWAS graphing with mouse clicks. BMC Bioinforma..

[44] Pruim RJ (2010). LocusZoom: regional visualization of genome-wide association scan results. Bioinformatics.

[45] Altman DG, Bland JM (2003). Interaction revisited: the difference between two estimates. BMJ.

[46] Pers TH (2015). Biological interpretation of genome-wide association studies using predicted gene functions. Nat. Commun..

