# Supplementary material for: Genetic architecture of type 1 diabetes with low genetic risk score informed by 41 unreported loci
Source: Commun Biol. 2021 Jul 23;4:908. doi: 10.1038/s42003-021-02368-8 (PMC8302754; doi:10.1038/s42003-021-02368-8)
Supplement: Supplementary file 1 — Description of Supplementary Files [file 42003_2021_2368_MOESM1_ESM.pdf]

Description of Additional Supplementary Files

File name: Supplementary Data 1

Description: SNP markers in the GRS2 system.

File name: Supplementary Data 2

Description: The ROC analysis of the GRS scores.

File name: Supplementary Data 3

Description: SNPs associated with both low GRS T1D and overall T1D with genome-wide significance.

File name: Supplementary Data 4

Description: SNPs in non-HLA regions associated with low GRS T1D with genome-wide significance.

File name: Supplementary Data 5

Description: Reported association by previous GWAS studies (GWAS Catalog, https://www.ebi.ac.uk/gwas/).
